# Interplay between lifestyle factors and polygenic risk for incident coronary heart disease in a large multiethnic cohort

**DOI:** 10.1016/j.ijcrp.2024.200350

**Published:** 2024-11-06

**Authors:** Carlos Iribarren, Meng Lu, Martha Gulati, Nathan D. Wong, Roberto Elosua, Jamal S. Rana

**Affiliations:** a Kaiser Permanente Northern California Division of Research, Oakland, CA, USA; b Barbra Streisand Women's Heart Center, Smidt Heart Institute, Cedars-Sinai Medical Center, Los Angeles, CA, USA; c Heart Disease Prevention Program, Division of Cardiology, Department of Medicine, University of California Irvine, Irvine, CA, USA; d Cardiovascular Epidemiology and Genetics, Institut Hospital del Mar d'Investigacions Mèdiques (IMIM), CIBER Cardiovascular Diseases (CIBERCV), Barcelona, Faculty of Medicine, University of Vic-Central University of Catalonia (UVic-UCC), Vic, Spain; e Department of Cardiology, The Permanente Medical Group, Kaiser Permanente Oakland Medical Center, Oakland, CA, USA

**Keywords:** Polygenic risk score, Coronary heart disease risk, Lifestyle factors

## Abstract

**Introduction:**

The objective of this study was to examine the interplay of polygenic risk and individual lifestyle factors (and a composite score of lifestyle) as antecedents of CHD in a large multiethnic cohort.

**Methods:**

We used Genetic Epidemiology Resource in Adult Health and Aging (GERA) cohort participants free of CHD at baseline (n = 60,568; 67 % female; 18 % non-European). The individual and joint associations of smoking, Mediterranean diet pattern, level of physical activity and polygenic risk with incident CHD were assessed using Cox regression adjusting for genetic ancestry and non-mediating risk factors. Hazard ratios (HRs) and number needed to treat (NNT) were estimated according to these lifestyle factors and polygenic risk categories. Strengths included large sample size, long-follow-up, ethnic diversity, a clinically-validated polygenic risk score (PRS), and rich phenotype information.

**Results:**

After 14 years of follow-up, there were 3159 incident CHD events. We observed no statistically significant interactions between individual lifestyle factors and polygenic risk (all p > 0.23). For individuals with a high genetic risk, moving from the worse lifestyle combination (no favorable lifestyle factors) to the best lifestyle combination (all three) is associated with 52 % lower rate of CHD. The NNT was highest in the low polygenic risk group (34), lowest in the high polygenic risk group [19] and in-between (Jin et al., 2011) [24] in the intermediate polygenic risk group.

**Conclusions:**

Lifestyle and polygenic risk together influence the risk of incident CHD. Our results support consideration of polygenic risk in lifestyle interventions because those with high polygenic risk are likely to derive the most benefit.

## 1 Introduction

Considerable progress has been made over the last decade unraveling the complex genetic architecture of coronary heart disease (CHD) [1]. A recent American Heart Association (AHA) Statement has recognized polygenic risk scores as useful tools for earlier screening for subclinical atherosclerosis and as a risk enhancing factor for primary prevention in middle-aged patients at borderline-intermediate 10-year atherosclerotic cardiovascular disease (ASCVD) [2]. However, few studies to date have examined the joint contribution of polygenic risk and lifestyle factors known to be major drivers behind CHD risk [[3], [4], [5], [6]].

The first report on this topic leveraged three large cohorts (the Atherosclerosis Risk in Communities Study, the Women's Genome Health Study and the Malmo Diet and Cancer Study) and concluded that a CHD polygenic risk score and a lifestyle score were independently associated with susceptibility to CHD [4]. Furthermore, among participants with high genetic risk, favorable lifestyle was associated with a 50 % lower relative risk relative to those with unfavorable lifestyle.

In this current study, our goal was to add to the literature on this topic by examining the interplay of polygenic risk and individual lifestyle factors (in addition to a composite score of lifestyle) as antecedents of CHD in a large multiethnic cohort. In particular, we reexamined the premise that the effect of high genetic risk is lower in the presence of favorable lifestyles.

## 2 Methods

### 2.1 Study cohort

The Genetic Epidemiology Resource in Adult Health and Aging (GERA) cohort included 110,266 adult male and female Kaiser Permanente of Northern California (KPNC) members who completed a self-administered questionnaire in 2007-08 and donated a saliva sample. Detailed information on recruitment and characteristics of the GERA cohort is available elsewhere [7]. Of those, 97,823 had complete genomic data for estimation of the PRS and principal components of genetic ancestry. Further sequential exclusions were age under 30 or age over 79) (n = 8416), prevalent CHD (n = 2481) and missing data on race/ethnicity, smoking, diet or physical activity (n = 26,358), resulting in a cohort of 60,568 subjects (Supplemental Fig. 1). We did not exclude subjects with missing education level (5.2 %) or BMI (2.8 %). The study received approval from the Kaiser Foundation Research Institute Institutional Review Board, and all subjects provided informed consent.

### 3.2 Genotyping

Genotyping was performed at the Institute for Human Genetics, University of California San Francisco, using custom-designed Affymetrix Axiom arrays as previously published [8]. The genome-wide arrays yielded high-quality genotypes, with an average genotype call rate of 99.7 % and SNP reproducibility of 99.9 % [9]. Details regarding the development and clinical validation of the polygenic risk score (CARDIO inCode-Score® CHD PRS, GENinCode US Inc.) can be found elsewhere [10]. The SNPs included in the CARDIO inCode-Score® CHD PRS underwent a meticulous selection process spanning over a decade [[10], [11], [12], [13], [14], [15], [16]]. This process started with genome-wide SNP panels identified by the CARDIoGRAMplusC4D Consortium [17], ultimately culminating in a refined 12 SNP panel [12]. All 12 SNPs are linked to CHD but operate independently from conventional risk factors like LDL cholesterol, HDL cholesterol, blood pressure, smoking, and diabetes mellitus. This independence sets them apart from the majority of SNPs in genome-wide PRSs. Developed by GENinCode Plc for primary preventive clinical use, the CARDIO inCode-Score® CHD PRS is readily available in Europe. Its effectiveness has been demonstrated in enhancing risk assessment, especially in multi-ethnic populations with intermediate ASCVD risk when integrated into the Framingham risk function and the Pooled Cohorts Equations. (10), (12) and (13) In the realm of secondary prevention, it has shown correlation with increased recurrence risk following a first myocardial infarction [15,16]. Notably, the CARDIO inCode-Score® represents a pioneering PRS, earning De Novo status from the FDA and is currently advancing through regulatory approval processes.

### 3.3 Lifestyle factors and covariates

Smoking status (never, former, current) was determined from responses to the baseline survey (2007-09). The Mediterranean diet pattern was constructed using items from a food frequency questionnaire capturing a diet rich in fruits, vegetables, fish, nuts and seeds plus limited consumption of red meat and processed meats (Appendix 1) [18]. We classified cohort members into meeting or not current physical activity recommendations for US adults *(*at least 150 min/week of moderate intensity exercise or 75 min/week or vigorous intensity exercise) [19] using self-reported physical activity measures (including brisk walking and recreational physical activity and physical labor) in the baseline survey. Questionnaire items and algorithms are provided in Appendix 2**.**

Information on age, sex, race/ethnicity, education level, body mass index (BMI, weight in kg/height in m^2^) and family history of heart attack (both parents, at any age) was available from the baseline survey. Systolic (SBP) and diastolic blood pressure (DBP) measurements were collected from primary care outpatient visits closest (and within 2 years) to the survey date. Hypertension was defined as SBP >140 mmHg or DBP >90 mmHg or use of antihypertensives. Diabetes status was determined by cross-referencing with the KPNC diabetes registry. The diabetes algorithm incorporates information from inpatient and outpatient diagnosis, laboratory values (A1c, fasting and post-load glucose), plus diabetes medication prescriptions [20]. Information regarding hypertension and hypercholesterolemia treatment was ascertained using the Pharmacy Information Management System (PIMS), which relies on prescription dispensing records of drugs belonging to the corresponding therapeutic class, obtained either at the time of the baseline survey or up to two years prior.

### 3.4 Study outcome

The primary outcome of interest was the occurrence of incident CHD from the baseline period (2007-09) through December 31, 2022. Ischemic stroke or heart failure were not included because the 12-SNP PRS was originally developed to predict CHD and no other CVD outcomes [14]. Incident CHD was ascertained using a hospital primary discharge diagnosis of myocardial infarction, angina or coronary atherosclerosis, coronary revascularization procedures (coronary bypass or percutaneous intervention), or death due to CHD. For completeness of event ascertainment, events that occurred outside of health plan hospitals were captured using claims data. The International Classification of Diseases, Ninth and Tenth Revisions (ICD-9 and ICD-10) codes were used for event ascertainment (Supplemental Table 1). The validity of these codes has been demonstrated in prior studies conducted within the KPNC population [21,22]. For angina or coronary atherosclerosis events occurring after 2014 and coded using ICD-10, evidence of significant coronary stenosis >50 % on angiography was required and confirmed through review of electronic medical records by one of the MD investigators (C.I.)

### 3.5 Statistical analyses

We described the characteristics of the cohort at baseline by calculating mean (SD) of continuous variables and reporting distributions of categorical variables. We first considered a main-effects Cox regression model to assess independent effects on incident CHD of former and never smoking, adhering to a Mediterranean diet pattern, meeting physical activity recommendations, polygenic risk categories (quintiles 2–4 and 5 vs quintile 1, respectively), plus covariates: demographic factors (age, sex, 10 principal components of ancestry), education level, use of cholesterol lowering drugs and family history of heart attack. Diabetes, hypertension and BMI were not entered into the model because of being downstream mediating traits from lifestyle factors. Censoring occurred after incident CHD, death, or disenrollment from the health plan [23]. In addition to the main effects model, we formally tested interactions between the PRS as a continuous variable and individual lifestyle factors. We then estimated age-adjusted rates of incident CHD according to individual lifestyle factors and, within levels of lifestyle factors, by low (quintile 1), medium (quintiles 2–4) and high (quintile 5) polygenic risk, as defined before [12]. Tests for linear trends were examined across PRS groups for each level of the corresponding lifestyle factor. Next, we assessed the joint association between the PRS and lifestyle factors with incident CHD using the Cox proportional hazards model. First, we considered lifestyles factors individually. The reference group was low polygenic risk and the corresponding lifestyle group with a *priory* lowest risk (never smokers, adhering to Mediterranean diet and meeting physical activity recommendations, respectively). The first model was adjusted for age, sex and 10 principal components of genetic ancestry [24], and the fully-adjusted model included additional covariates for education level, use of lipid-lowering drugs and family history of heart attack. We did not adjust for downstream risk factors (BMI, diabetes, hypertension, hyperlipidemia) because of being in the causal pathway of lifestyle factors. Next, we constructed a 3-level categorical variable for overall lifestyle: level “0” if the three favorable lifestyles were present (i.e., never smoking, Mediterranean diet and meeting physical activity recommendations); level “1” if one or any combination of two lifestyle factors were present; and “2” if none favorable lifestyle factors were present. In this scenario, we employed two different approaches. First, a single model having a single reference group defined as low polygenic risk plus having three favorable risk factors. Second, three separate models, one for each polygenic risk group, with having 3 favorable risk factors as reference. In addition, we formally tested, in a fully-adjusted model (as described above) the interaction of the combined categorical lifestyle variable with the PRS as a continuous variable. Finally, to evaluate the effectiveness of the PRS, we estimated the numbers needed to treat (NNT) to prevent 1 CHD event [25] for a theoretical intervention of moving someone from 3 unfavorable lifestyles to 0 unfavorable lifestyles, separately for individuals in the low, intermediate and high polygenic risk groups.

## 3 Results

At baseline, the cohort had a mean (SD) age of 59 [9] years, and 67 % of the participants were female (Table 1). The higher percentage of women (67 %) reflects their greater participation in the RPGEH survey. The majority of the cohort, approximately 82 %, self-identified as European, while 3 % self-identified as African-American, 7 % as Hispanic/Latino, and 7 % as Asian. About 81 % of the study participants had a college education or higher, 5 % reported being current smokers, while 36 % were former smokers. Whereas 23 % followed a Mediterranean Diet pattern, 38 % met the recommended amount of physical activity. The prevalence of diabetes was 14 %, and 24 % had a BMI within the obesity range. Approximately 49 % of the participants had hypertension, and about one third were using lipid-lowering therapy. Additionally, 29 % of the participants reported a family history of heart attack.

**Table 1:** Characteristics of the GERA cohort (n = 60,568).

Age at Survey, years (mean ± SD)	58.7 (9.41)
30–54	19086 (31.5 %)
55–64	22690 (37.5 %)
65–79	18792 (31.0 %)
Gender, n (%)	
Male	19782 (32.7 %)
Female	40786 (67.3 %)
Race/ethnicity, n (%)	
White	49889 (82.4 %)
African-American	1984 (3.3 %)
Hispanic/Latino	4141 (6.8 %)
Asian	4554 (7.5 %)
Education level, n (%)	
Less than college	8192 (13.5 %)
College or higher	49234 (81.3 %)
Missing	3142 (5.2 %)
Smoking status, n (%)	
Never	35752 (59.0 %)
Former	21847 (36.1 %)
Current	2969 (4.9 %)
Body mass index, kg/m^2^ (mean ± SD)	27.2 (5.68)
< 25	23601 (39.0 %)
25–29.9	20898 (34.5 %)
≥30	14380 (23.7 %)
Missing	1689 (2.8 %(
Mediterranean Diet Pattern, n (%)	
Yes	14216 (23.5 %)
No	46352 (76.5 %)
Meeting Physical Activity Recommendations, n (%)	
Yes	23375 (38.6 %)
No	37193 (61.4 %)
Diabetes mellitus, n (%)	8284 (13.7 %)
Hypertension, n (%)	29463 (48.6 %)
Cholesterol lowering drugs, n (%)	20281 (33.5 %)
Family history of heart attack, n (%)	17896 (29.5 %)

After a mean (SD) follow-up time of 14 [4] years, 3159 incident CHD events were documented. In multivariable Cox regression analysis in the entire cohort, former and current smoking were associated with 1.14-fold and 1.48-increased hazard of CHD, respectively (Table 2). Adhering to a Mediterranean diet was associated with 14 % reduction in the hazard of CHD, whereas meeting physical activity recommendations was associated with a 15 % reduction in the hazard of CHD. In turn, PRS in quintiles 2–4 and 5 were associated with 1.27 and 1.68-fold increased hazard of CHD, respectively. We also noted significant increased hazard of CHD with age, male sex, use of cholesterol lowering drugs and family history of heart attack (Table 2). None of the interactions between continuous PRS and individual lifestyle factors were statistically significant (all p > 0.23).

**Table 2:** Main independent effects model of incident CHD in the GERA cohort (n = 60,568).

Main Independent Effects Adjusting for 10 ancestry PC	HR (95 % CI)	p
Age, per 1 SD	1.82 (1.73–1.90)	<0.001
Male sex, vs. female sex	2.47 (2.29–2.66)	<0.001
Less than a College Education vs. College or Higher	1.30 (1.18–1.43)	<0.001
Former Smoking vs. Never	1.14 (1.05–1.22)	<0.001
Current Smoking vs. Never	1.48 (1.28–1.71)	<0.001
Mediterranean Diet (Yes vs. No)	0.86 (0.78–0.94)	<0.001
Meeting Physical Activity Recommendations (Yes vs. No)	0.85 (0.79–0.91)	<0.001
PRS Quintiles 2, 3 and 4 vs. 1	1.27 (1.16–1.41)	<0.001
PRS Quintile 5 vs. 1	1.68 (1.50–1.88)	<0.001
Cholesterol lowering drug use (Yes vs. No)	2.04 (1.90–2.20)	<0.001
Family history of heart attack (Yes vs. No)	1.48 (1.37–1.59)	<0.001

**Fig. 1:**
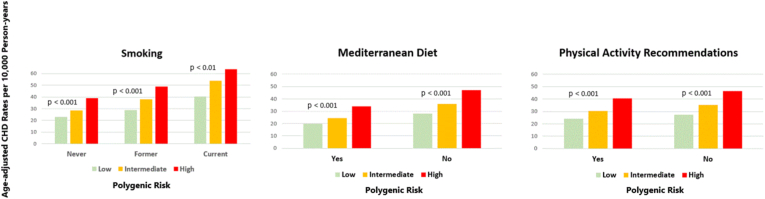
Age-adjusted rates of CHD by joint categories of individual lifestyle factors and polygenic risk.

There were consistent statistically significant positive linear trends of age-adjusted rates per 10,000 person-years of follow-up of incident CHD by polygenic risk in each level of individual lifestyle factors (Fig. 1).

Results of a single multivariate Cox regression model examining joint categories of individual lifestyle factors and low (first quintile)/intermediate (quintiles 2–4)/high (quintile 5) polygenic risk are provided in Table 3. For each lifestyle factor, the most favorable level (never smokers, following Mediterranean diet pattern or meeting physical activity recommendations, respectively) plus being in the low genetic risk group (first quintile) was the reference level. Current smokers with high genetic risk were at 2.4-fold higher risk of CHD compared with never smokers with low genetic risk. Not following a Mediterranean diet pattern and being in the high genetic risk group was associated with 2-fold increased risk of CHD compared with following a Mediterranean diet pattern and being in the low genetic risk group. Not meeting physical activity recommendations and being in the high genetic risk group was also associated with 2-fold increased risk of CHD compared with meeting physical activity recommendations and being in the low genetic risk group.

**Table 3:** Adjusted∗ Hazard Ratios (95 % Confidence Intervals) of Incident CHD by Joint Categories of Individual Lifestyle Factors and Polygenic Risk in the GERA Cohort (n = 60,568). ∗10 principal components of ancestry, age, sex, education level and family history of heart disease.

Individual Lifestyles	Polygenic Risk											
	Low				Intermediate				High			
	Num subjects	Num events	AHR (95 % CI)	p	Num subjects	Num events	AHR (95 % CI)	p	Num subjects	Num events	AHR (95 % CI)	p
Smoking Status												
Never	7257	241	1.00		22117	895	1.23 (1.07–1.42)	0.01	4032	342	1.97 (1.67–2.32)	<0.001
Former	4427	230	1.13 (0.94–1.35)	0.19	13388	888	1.50 (1.30–1.73)	<0.001	1862	133	1.96 (1.59–2.43)	<0.001
Current	580	32	1.37 (0.95–1.99)	0.09	1862	133	1.96 (1.59–2.43)	<0.001	527	45	2.39 (1.74–3.29)	<0.001
Mediterranean Diet Pattern												
Yes	2856	96	1.00		8735	352	1.27 (1.01–1.59)	0.04	2625	148	1.80 (1.39–2.33)	<0.001
No	9408	407	1.21 (0.97–1.51)	0.09	28623	1564	1.56 (1.27–1.92)	<0.001	8312	592	2.06 (1.66–2.56)	<0.001
Meeting Physical Activity Recommendations												
Yes	4705	179	1.00		14325	676	1.26 (1.07–1.48)	0.01	4345	279	1.65 (1.37–2.00)	<0.001
No	7559	324	1.19 (0.99–1.42)	0.06	23042	1240	1.54 (1.31–1.80)	<0.001	6592	461	2.03 (1.71–2.42)	<0.001

Next, we considered a single model examining combined lifestyle factors where the reference group was having 3 favorable lifestyle factors and being in the low polygenic risk group (Table 4, upper panel). There was a clear gradation of risk in each polygenic risk group according to worsening lifestyle. Those with the least favorable lifestyle combination (no favorable lifestyle factors) and high genetic polygenic risk were at 3.4-fold risk of CHD compared to the reference group. In the complimentary approach of examining the effect of combined lifestyle factors separately in each polygenic risk group (Table 4, lower panel), we observed similar associations of worsening lifestyle with incident CHD across polygenic risk groups. In a fully adjusted model, the interaction of PRS as a continuous variable and the 3-level combined lifestyle variable was not statistically significant (p = 0.11).

**Table 4:** Adjusted^a^ hazard ratios (95 % confidence intervals) of incident CHD by joint categories of combined lifestyle factors and polygenic risk in the GERA cohort (n = 60,568).

	Single model where reference group is low polygenic risk and having three favorable lifestyle factors											
	Polygenic Risk											
	Low				Intermediate				High			
Combined Lifestyles∗	Num subjects	Num events	AHR† (95 % CI)	p	Num subjects	Num events	AHR (95 % CI)	p	Num subjects	Num events	AHR (95 % CI)	p
3 favorable	7257	241	1.00		22117	895	1.31 (0.82–2.10)	0.25	6378	353	1.65 (0.96–2.82)	0.07
1 or 2 favorable	4427	230	1.37 (0.90–2.11)	0.14	13388	888	1.75 (1.15–2.67)	0.01	4032	342	2.33 (1.52–3.56)	<0.001
0 favorable	580	32	2.20 (1.23–3.93)	0.01	1862	133	2.97 (1.87–4.73)	<0.001	527	45	3.37 (1.94–5.84)	<0.001

Three models stratifying by polygenic risk and where the reference group is having 3 favorable lifestyle factors												
	**Low**				**Intermediate**				**High**			

3 favorable	7257	241	1.00		22117	895	1.00		6378	353	1.00	
1 or 2 favorable	4427	230	1.31 (0.85–2.02)	0.22	13388	888	1.26 (1.01–1.57)	0.04	4032	342	1.29 (0.91–1.83)	0.15
0 favorable	580	32	1.58 (1.01–2.49)	0.04	1862	133	1.74 (1.38–2.18)	<0.001	527	45	1.84 (1.28–2.64)	0.01

†10 principal components of ancestry, age, sex, education level and family history of heart disease.

a 3 favorable = never smoking + Mediterranean pattern diet + meeting physical activity recommendations; 1 or 2 favorable = any single favorable lifestyle factor or any combination of 2 favorable lifestyle factors; 0 favorable = current smoking + no Mediterranean pattern diet + not meeting physical activity recommendations.

**Fig. 2:**
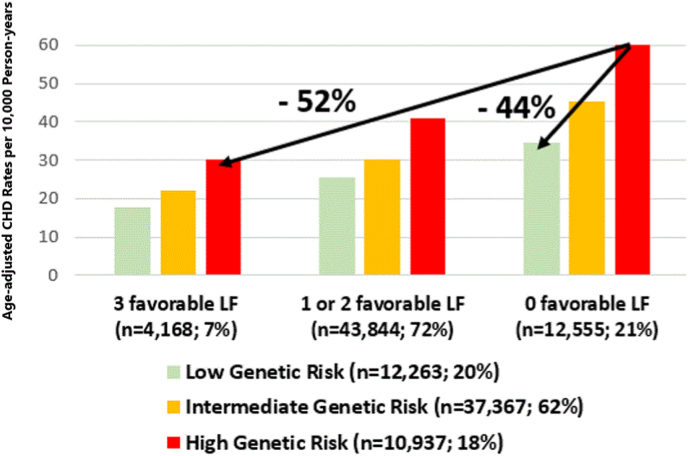
Age-adjusted rates of CHD by joint categories of combined lifestyle factors and polygenic risk.

As Fig. 2 demonstrates, for individuals with a high genetic risk, moving from the worse lifestyle combination (no favorable lifestyle factors) to the best lifestyle combination (all three) translated into a 52 % lower rate of CHD. On the other hand, among subjects with the worse lifestyle combination, moving from high to low polygenic risk translated into a 44 % lower rate of CHD.

Finally, we considered a theoretical scenario where the “experimental arm” was having 3 favorable lifestyle factors and the “control or placebo arm” was having 0 favorable lifestyle factors and calculated the Numbers Needed to Treat (NNT) to prevent 1 CHD, separately in each polygenic risk group. The NNT was highest in the low polygenic risk group (34), lowest in the high polygenic risk group [19] and in-between [24] in the intermediate polygenic risk group.

## 4 Discussion

This analysis in a large-multiethnic cohort in a real-world contemporary setting highlights the importance of both lifestyle and inherited genetic risk of CHD. The most salient findings are: 1) a clear gradation of CHD risk according to lifestyle at every level of polygenic risk; 2) among those with high genetic risk, moving from the worse to the best lifestyle combination translates into a 52 % reduction in the rate of incident CHD; and 3) overall, the NNT to prevent 1 CHD event was much lower in the high genetic risk group [19] compared with the low genetic risk group (34). The primary reason for the lower NNT in the high genetic risk group is largely the higher absolute risk that is conferred by the polygenic risk. This suggests that lifestyle interventions, although benefit everyone, would be more effective among individuals with high genetic burden for CHD.

Despite methodological differences, our results replicate those of Khera et al. using data from 3 cohorts (n = 55,685) concluding that, among participants with high genetic risk, favorable lifestyle was associated with a 50 % lower relative risk relative to those with unfavorable lifestyle [4]. One of the main difference was the consideration of obesity as a lifestyle factor in prior studies. Unlike Khera et al. we did not include BMI as a lifestyle factor because, rather than being a lifestyle factor, it is a downstream phenotype resulting from the interaction of genetic predisposition, the environment and health behaviors. Our results are also in agreement with a report by Said et al. using the UK Biobank cohort (N = 339,003) investigating the combined effects of lifestyle and genetic risk factors on CHD outcomes [5]. Lifestyle factors were defined as smoking, BMI, physical activity and diet. Results of this study showed an additive effect between the risk of developing CHD, combined health behaviors and genetic risk. More recently, Ye et al., also using the UK Biobank, concluded that individuals with high genetic risk may derive similar relative but greater absolute benefit from lifestyle adherence [26]. Our results are also congruent with the findings of Livingstone et al. demonstrating benefits of healthy diet regardless of genetic risk [27] and with the study of Tikkanen et al. showing that higher grip strength and cardiorespiratory fitness were associated with lower risk of incident CHD in each genetic risk score group [28]. On the other hand, our results of no significant interaction between polygenic risk and smoking are at odds with the report of a significant interaction between these two factors in the Malmö Diet and Cancer Study [29]. It should be pointed out that the prevalence of current smoking was much higher in the Malmö Study (28 %) than in ours (5 %).

Our study has several strengths, including the availability of a large, U.S. based ethnically diverse cohort, with two-thirds of our sample being female, and followed for an average of 14 years. Rather than a health maintenance organization (HMO, claims data), KPNC is an integrated health care delivery system where utilization comes from the systems own hospitals, outpatient clinics, a central laboratory and pharmacies. As long as members remain in the plan, ascertainment of inpatient services is essentially complete. Among persons in the GERA cohort, over 97 % have at least 5 years of continuous membership, and over 83 % have at least 10 years of continuous membership with an average duration of membership of 23.5 years [7]. In addition, the PRS used in the current study (CARDIO inCode-Score®) has been clinically valida ted [30], is commercially available, and is a relatively simple technology with a short turnaround time. We also recognize some limitations in our study. First, our cohort participants were all members of KPNC, therefore findings may not fully generalize to uninsured or other populations. Moreover, the GERA cohort has a high representation of the upper end of the educational spectrum, which could limit its generalizability to populations with lower educational levels. Second, the PRS was developed and validated using European-based genetic panels and thus is not fully optimized for African American or Asian subjects. Third, we did not consider the American Heart Association (AHA) Life's Essential 8 score because the GERA cohort does not have information on sleep health. Finally, as with any study involving lifestyle factors, there are inherent biases due to self-reported data. Variables such as diet, exercise, and smoking status are subject to change over time, and self-reported information from 2007/08 may not accurately reflect the participants' habits throughout the study period.

A recent scientific statement from the AHA concludes that the CAD PRS can provide additional prognostic information that may have utility in guiding pharmacological management, particularly for LDL-C lowering [2]. Moreover, CAD PRSs can identify younger individuals who may benefit the most from more aggressive lifestyle modification [4,31]. Accordingly, knowledge of the PRS can motivate individuals to make extensive lifestyle changes, similar to that seen in CAC scoring [32]. Additional benefit is that, unlike CAC scoring, PRS can be tested at a much younger age with ability to motivate and modify lifestyle factors upstream.

In summary, lifestyle and polygenic risk have additive effects on the risk of incident CHD. Moreover, our results support consideration of PRS in lifestyle interventions because those with high polygenic risk are more likely to derive benefit.

## Funding

This study was funded by a grant to Dr. Iribarren by GENinCode, Plc.

## CRediT authorship contribution statement

**Carlos Iribarren:** Writing – original draft, Visualization, Validation, Supervision, Resources, Project administration, Methodology, Investigation, Funding acquisition, Data curation, Conceptualization. **Meng Lu:** Writing – review & editing, Software, Formal analysis, Data curation. **Martha Gulati:** Writing – review & editing. **Nathan D. Wong:** Writing – review & editing. **Roberto Elosua:** Writing – review & editing. **Jamal S. Rana:** Writing – review & editing.

## Conflict of interest disclosures

Carlos Iribarren received a research grant from GENinCode, Plc, for this study.

Meng Lu has no disclosures.

Martha Gulati serves on the advisory Board for Esperion, Novartis and Boehringer Ingelheim.

Nathan Wong receives research support through his institution from Novartis, Novo Nordisk, and Regeneron and is a consultant for Novartis and Ionis.

Roberto Elosua is a member of the scientific advisory board of GENinCode, Plc, and inventor in a patent application based on the CARDIOinCODE-Score® CHD PRS whose applicant is GENinCode, Plc.

Jamal S. Rana has no disclosures.

## References

[1] Chen Z., Schunkert H. (2021). Genetics of coronary artery disease in the post-GWAS era. J. Intern. Med..

[2] O'Sullivan J.W., Raghavan S., Marquez-Luna C. (2022). Polygenic risk scores for cardiovascular disease: a scientific statement from the American heart association. Circulation.

[3] Hasbani N.R., Ligthart S., Brown M.R. (2022). American heart association's life's simple 7: lifestyle recommendations, polygenic risk, and lifetime risk of coronary heart disease. Circulation.

[4] Khera A.V., Emdin C.A., Drake I. (2016). Genetic risk, adherence to a healthy lifestyle, and coronary disease. N. Engl. J. Med..

[5] Said M.A., Verweij N., van der Harst P. (2018). Associations of combined genetic and lifestyle risks with incident cardiovascular disease and diabetes in the UK Biobank study. JAMA Cardiol.

[6] Wang M., Brage S., Sharp S.J., Luo S., Au Yeung S.L., Kim Y. (2022). Associations of genetic susceptibility and healthy lifestyle with incidence of coronary heart disease and stroke in individuals with hypertension. Eur J Prev Cardiol.

[7] Banda Y., Kvale M.N., Hoffmann T.J. (2015). Characterizing race/ethnicity and genetic ancestry for 100,000 subjects in the genetic Epidemiology research on adult health and aging (GERA) cohort. Genetics.

[8] Hoffmann T.J., Zhan Y., Kvale M.N. (2011). Design and coverage of high throughput genotyping arrays optimized for individuals of East Asian, African American, and Latino race/ethnicity using imputation and a novel hybrid SNP selection algorithm. Genomics.

[9] Hoffmann T.J., Kvale M.N., Hesselson S.E. (2011). Next generation genome-wide association tool: design and coverage of a high-throughput European-optimized SNP array. Genomics.

[10] Iribarren C., Lu M., Elosua R. (2024). Polygenic risk and incident coronary heart disease in a large multiethnic cohort. Am J Prev Cardiol.

[11] Executive Summary of The Third Report of The National Cholesterol Education Program (NCEP) (2001). Expert panel on detection, evaluation, and treatment of high blood cholesterol in adults (adult treatment panel III). JAMA.

[12] Iribarren C., Lu M., Jorgenson E. (2016). Clinical utility of multimarker genetic risk scores for prediction of incident coronary heart disease: a cohort study among over 51 thousand individuals of European ancestry. Circ Cardiovasc Genet.

[13] Iribarren C., Lu M., Jorgenson E. (2018). Weighted multi-marker genetic risk scores for incident coronary heart disease among individuals of african, Latino and east-asian ancestry. Sci. Rep..

[14] Lluis-Ganella C., Subirana I., Lucas G. (2012). Assessment of the value of a genetic risk score in improving the estimation of coronary risk. Atherosclerosis.

[15] Rincon L.M., Sanmartin M., Alonso G.L. (2020). A genetic risk score predicts recurrent events after myocardial infarction in young adults. Rev. Esp. Cardiol..

[16] Rincon L.M., Subirana I., Perez Del Villar C. (2023). Predictive capacity of a genetic risk score for coronary artery disease in assessing recurrences and cardiovascular mortality among patients with myocardial infarction. Front Cardiovasc Med.

[17] Deloukas P., Kanoni S., Willenborg C. (2013). Large-scale association analysis identifies new risk loci for coronary artery disease. Nat. Genet..

[18] Estruch R., Ros E., Salas-Salvado J. (2018). Primary prevention of cardiovascular disease with a mediterranean diet supplemented with extra-virgin olive oil or nuts. N. Engl. J. Med..

[19] Services USDoHaH (2018).

[20] Karter A.J., Schillinger D., Adams A.S. (2013). Elevated rates of diabetes in pacific islanders and asian subgroups: the diabetes study of northern California (DISTANCE). Diabetes Care.

[21] Iribarren C., Chandra M., Lee C. (2022). Breast arterial calcification: a novel cardiovascular risk enhancer among postmenopausal women. Circ Cardiovasc Imaging.

[22] Selby J.V., Fireman B.H., Lundstrom R.J. (1996). Variation among hospitals in coronary-angiography practices and outcomes after myocardial infarction in a large health maintenance organization. N. Engl. J. Med..

[23] Cox D.R. (1972). Regression models and life-tables. J R Stat Soc [B].

[24] Jin J., Cerise J.E., Kang S.J. (2011). Principal components ancestry adjustment for Genetic Analysis Workshop 17 data. BMC Proc..

[25] Laupacis A., Sackett D.L., Roberts R.S. (1988). An assessment of clinically useful measures of the consequences of treatment. N. Engl. J. Med..

[26] Ye Y., Chen X., Han J., Jiang W., Natarajan P., Zhao H. (2021). Interactions between enhanced polygenic risk scores and lifestyle for cardiovascular disease, diabetes, and lipid levels. Circ Genom Precis Med.

[27] Livingstone K.M., Abbott G., Bowe S.J., Ward J., Milte C., McNaughton S.A. (2021). Diet quality indices, genetic risk and risk of cardiovascular disease and mortality: a longitudinal analysis of 77 004 UK Biobank participants. BMJ Open.

[28] Tikkanen E., Gustafsson S., Ingelsson E. (2018). Associations of fitness, physical activity, strength, and genetic risk with cardiovascular disease: longitudinal analyses in the UK Biobank study. Circulation.

[29] Hindy G., Wiberg F., Almgren P., Melander O., Orho-Melander M. (2018). Polygenic risk score for coronary heart disease modifies the elevated risk by cigarette smoking for disease incidence. Circ Genom Precis Med.

[30] Iribarren C LM, Elosua R, Gulati M, Wong ND, Blumenthal RS, Nissen S, Rana JS. . Polygenic Risk and Incident Coronary Heart Disease in a Large Multiethnic Cohort Am J Preventive Cardiol in press. 10.1016/j.ajpc.2024.100661 PMC11004687 38601895

[31] Iribarren C.L.M., Elosua R., Rana J.S. (2023). Utility of a polygenic risk score for incident CHD: interplay with lifestyle in a multi-ethnic cohort of more than 60,000 individuals. European Heart Journal Supplement.

[32] Gupta A., Lau E., Varshney R. (2017). The identification of calcified coronary plaque is associated with initiation and continuation of pharmacological and lifestyle preventive therapies: a systematic review and meta-analysis. JACC Cardiovasc Imaging.

