# Supplementary material for: Interplay between lifestyle factors and polygenic risk for incident coronary heart disease in a large multiethnic cohort
Source: Int J Cardiol Cardiovasc Risk Prev. 2024 Nov 6;23:200350. doi: 10.1016/j.ijcrp.2024.200350 (PMC11584587; doi:10.1016/j.ijcrp.2024.200350)
Supplement: Multimedia component 1 [file mmc1.docx]

**Supplemental Table 1.** Codes for Identification of CHD Events.

| Condition | Primary Hospital Discharge Diagnosis  ICD-9 Codes | ICD-9 Procedure Codes | CPT4 Codes | Primary Hospital Discharge Diagnosis Codes or Underlying Cause of Death  ICD-10 Codes | IC D-10 Procedure Codes |
| --- | --- | --- | --- | --- | --- |
| Unstable and stable angina pectoris | 411.1, 413.0, 413.1, 413.9 |  |  | I20.0, I24.0, I25.110, I25.119, I25.81, I25.7x |  |
| Acute myocardial infarction | 410.x |  |  | I21.x |  |
| Coronary revascularization procedure |  | 36.01, 36.02, 36.05, 36.06, 36.07, 36.09  36.10, 36.11, 36.12, 36.13, 36.14, 36.15, 36.16, 36.17, 36.19, 36.03 | 33510, 33511, 33512, 33513, 33514, 33515, 33516, 33517, 33518, 33519, 33521, 33522, 33523, 33530, 33533, 33534, 33535, 33536,  92980, 92981, 92982, 92984, 92995, 92996 |  | 021xxxx |
| Coronary heart disease death |  |  |  | I20-I22, I25 |  |


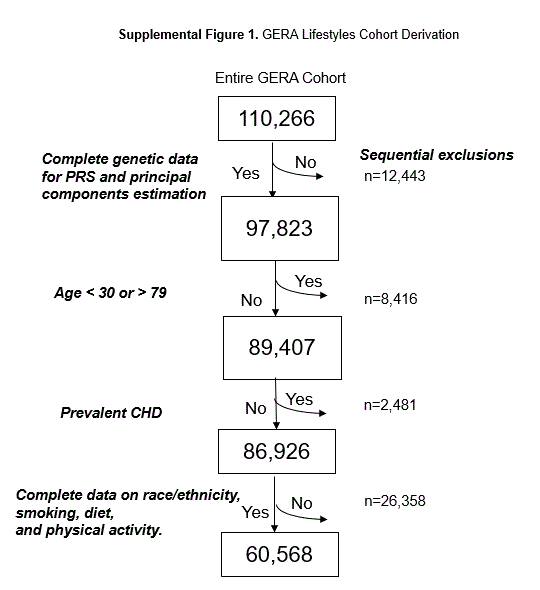


**Appendix 1.**


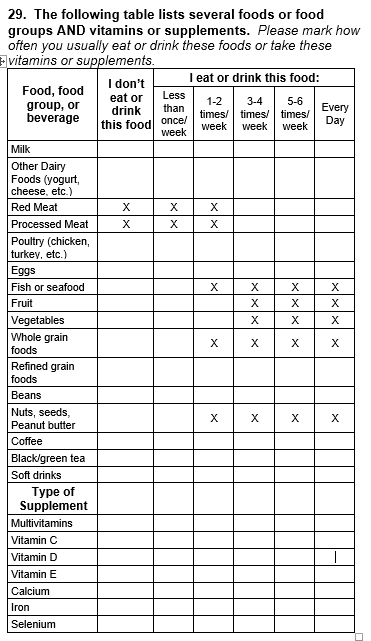


**Mediterranean Diet Pattern:** The following responses to the 5 food groups listed below are all required in order for a person to adhere to a Mediterranean Diet pattern.

Red meat or processed meats < 3 times/week +

Fish or seafood ≥ 1-2 times/week

+

Fruit and vegetables ≥ 3-4 times/week

+

Whole grain foods ≥ 1-2 times/week

+

Nuts, seeds ≥ 1-2 times per week.

**Appendix 2. RPGEH Physical Activity Questions.**

**31. During the past 7 days, on how many days did you walk briskly (at least 20 minute miles or fast enough to cause your heart rate to increase somewhat) for at least 10 minutes at a time? This includes at work and at home, walking to travel from place to place or any other walking that you might do for recreation, sport, or exercise? __** days per week

🡺 **IF no walking, skip to question 32**

**a. On average, how much time did you spend walking briskly each day you walked?**

__ __:__ __ each day you walked

hrs mins

**32. During the past 7 days, on how many days did you do recreational physical activity or physical labor for at least 10 minutes at a time that was vigorous enough to work up a sweat or cause your heart rate to increase substantially? __** days per week

🡺 **IF no vigorous activity, skip to question 33**

**a. On average, how much time did you spend doing vigorous physical activity each day you did it?**

__ __:__ __ each day you did vigorous physical activity

hrs min.

U.S. Department of Health and Human Services. Physical Activity Guidelines for Americans, 2nd edition (2018)

*For substantial health benefits, adults should do at least 150 minutes (2 hours and 30 minutes) to 300 minutes (5 hours) a week of moderate-intensity, or 75 minutes (1 hour and 15 minutes) to 150 minutes (2 hours and 30 minutes) a week of vigorous-intensity aerobic physical activity, or an equivalent combination of moderate- and vigorous-intensity aerobic activity. Preferably, aerobic activity should be spread throughout the week.*

Algorithm to determine meeting physical activity recommendations

Step 1: determine in each subject **minutes per week spent in moderate physical activity** (Q31 above; “*brisk walk*”): days per week x average minutes each day.

Step 2: determine in each subject **minutes per week spent in vigorous physical activity** (Q32 above; “*work up a sweat or cause heart rate to increase substantially*”): days per week x average minutes each day: days per week x average minutes each day.

Step 3: allocating subjects to “Yes” or “No” meeting physical activity recommendations:

If minutes per week spent in moderate physical activity > 150 then meeting physical activity recommendations = Yes

If minutes per week spent in vigorous physical activity > 75 then meeting physical activity recommendations = Yes

If minutes per week spent in moderate physical activity ≤ 150 but the sum of minutes per week spent on moderate and vigorous physical activity > 150 then physical activity recommendations = Yes

If minutes per week spent in vigorous physical activity ≤ 75 but the sum of minutes per week spent on moderate and vigorous physical activity > 150 then physical activity recommendations = Yes

Otherwise (none of the conditions met), then physical activity recommendations = No
